# Co-silencing of tomato *S*-adenosylhomocysteine hydrolase genes confers increased immunity against *Pseudomonas syringae* pv. *tomato* DC3000 and enhanced tolerance to drought stress

**DOI:** 10.3389/fpls.2015.00717

**Published:** 2015-09-08

**Authors:** Xiaohui Li, Lei Huang, Yongbo Hong, Yafen Zhang, Shixia Liu, Dayong Li, Huijuan Zhang, Fengming Song

**Affiliations:** National Key Laboratory for Rice Biology, Institute of Biotechnology, Zhejiang UniversityHangzhou, China

**Keywords:** tomato, *S*-adenosylhomocysteine hydrolase, disease resistance, drought tolerance

## Abstract

*S*-adenosylhomocysteine hydrolase (SAHH), catalyzing the reversible hydrolysis of *S*-adenosylhomocysteine (SAH) to adenosine and homocysteine, is a key enzyme that maintain the cellular methylation potential in all organisms. We report here the biological functions of tomato SlSAHHs in stress response. The tomato genome contains three *SlSAHH* genes that encode SlSAHH proteins with high level of sequence identity. qRT-PCR analysis revealed that *SlSAHHs* responded with distinct expression induction patterns to *Pseudomonas syringae* pv. *tomato* (*Pst*) DC3000 and *Botrytis cinerea* as well as to defense signaling hormones such as salicylic acid, jasmonic acid and a precursor of ethylene. Virus-induced gene silencing-based knockdown of individual *SlSAHH* gene did not affect the growth performance and the response to *Pst* DC3000. However, co-silencing of three *SlSAHH* genes using a conserved sequence led to significant inhibition of vegetable growth. The *SlSAHH*-co-silenced plants displayed increased resistance to *Pst* DC3000 but did not alter the resistance to *B. cinerea*. Co-silencing of *SlSAHHs* resulted in constitutively activated defense responses including elevated SA level, upregulated expression of defense-related and PAMP-triggered immunity marker genes and increased callose deposition and H_2_O_2_ accumulation. Furthermore, the *SlSAHH*-co-silenced plants also exhibited enhanced drought stress tolerance although they had relatively small roots. These data demonstrate that, in addition to the functions in growth and development, SAHHs also play important roles in regulating biotic and abiotic stress responses in plants.

## Introduction

*S*-adenosylhomocysteine hydrolase (SAHH) is a key enzyme in the activated methyl cycle and catalyzes the reversible hydrolysis of *S*-adenosylhomocysteine (SAH) to adenosine and homocysteine (Palmer and Abeles, 1979). Homocysteine is further converted to methionine and then to *S*-adenosylmethionine (SAM), which is a major methyl donor in transmethylation reactions. Accompanying the transfer of the activated methyl group of SAM via the transmethylation reactions to acceptors (e.g., phospholipids, proteins, DNA and RNA) is the formation of SAH, which is in turn a competitive inhibitor for almost all methyltransferases required for the transmethylation reactions (Chiang et al., 1996; Chiang, 1998). The SAHH-catalyzed conversion of SAH into adenosine and L-homocysteine can release the SAH-caused feedback inhibition, which can promote further continual transmethylation reactions. SAHH is therefore believed to play an important role in maintenance of the methylation potential for all biological systems through regulating the intracellular SAH/SAM ratio.

The biological function of SAHH in animals has been studied in detail using specific inhibitors and genetic mutants. Many diseases were found to be associated with changes in SAHH function in animals (Matthews et al., 2009). In higher plants, SAHHs with high affinity for methylation cofactors have been purified from tobacco and *Lupinus* (Sebestova et al., 1984; Brzezinski et al., 2008). Direct evidence supporting the biological functions of SAHHs in plant growth and development came from recent genetic and biochemical studies using loss-of-function and gain-of-function mutants. In *Arabidopsis*, there are two genes encoding for SAHHs, *AtSAHH1* (At4g13940) and *AtSAHH2* (At3g23810) (Rocha et al., 2005; Pereira et al., 2007; Li et al., 2008). *AtSAHH1* seems to be more important than *AtSAHH2* because mutations in *AtSAHH1* are embryonic lethal while mutations in *AtSAHH2* are morphologically indistinguishable from wild type (Rocha et al., 2005). Indeed, even partial loss of *AtSAHH1* function promotes developmental abnormalities such as delayed seed germination, slow growth, reduced size, low fertility and short primary roots with little or no root hairs (Furner et al., 1998; Rocha et al., 2005; Wu et al., 2009). Tobacco plants with reduced *SAHH* gene expression due to antisense inhibition in transgenic plants or reduced SAHH activity by specific inhibitor treatment are stunted, lack apical dominance and have floral abnormalities (Tanaka et al., 1997; Fulneček et al., 2011). Genome-wide analyses of gene expression and DNA methylation status in *Arabidopsis SAHH* mutant plants identified a large set of differentially expressed genes that are involved in pathways essential to plant growth and development and revealed DNA hypomethylation that is associated with gene silencing capabilities (Mull et al., 2006; Jordan et al., 2007; Li et al., 2008; Ouyang et al., 2012).

Several lines of evidence have also indicated that SAHHs play a role in plant response to pathogen infection. It was recently reported that betasatellite-encoded pathogenicity factor of *Tomato Yellow Leaf Curl China Virus*, βC1, and coat protein of *Tomato Chlorosis Virus* interact with SAHH, suppress its enzymatic activity and methylation-mediated transcriptional gene silencing in host plants (Yang et al., 2011; Cañizares et al., 2013). Transgenic tobacco plants with reduced *SAHH* gene expression showed less viral replication and increased resistance to infection by various viruses including *Tobacco Mosaic Virus, Potato Virus X*, and *Potato Virus Y* (Masuta et al., 1995). Differential expression of *SAHH* genes was observed recently in potato leaves after inoculation with *Phytophthora infestans* (Arasimowicz-Jelonek et al., 2013). The expression of *SAHH* gene was significantly upregulated in compatible interaction, whereas the expression was downregulated in incompatible interactions (Arasimowicz-Jelonek et al., 2013). Similarly, treatment with an elicitor derived from *Phytophthora megasperma* f. sp. *glycinea* strongly induced the expression of a *SAHH* gene in cultured cells as well as intact leaves of parsley (Kawalleck et al., 1992). These observations indicate a link between SAHHs and defense responses in plants.

The present study was aimed to explore the biological function of SAHHs in defense response in tomato against *Pseudomonas syringae* pv. *tomato* (*Pst*) DC3000, a (hemi)biotrophic bacterial pathogen, and *Botrytis cinerea*, a typical necrotrophic fungal pathogen and our data provide direct evidence supporting that, in addition to the previously reported functions in plant growth and development, SAHHs play important roles in regulating biotic and abiotic stress responses in plants.

## Materials and Methods

### Plant Growth Condition

Tomato (*Solanum lycopersicum*) cv. Suhong 2003 was used for all experiments. Seedlings were grown a mixture of perlite: vermiculite: plant ash (1:6:2) in a growth room under fluorescent light (200 μE m^2^ s^-1^) at 22–24°C with a 14 h light/10 h dark cycle. The relative humidity in the growth room was controlled around 60%. Two-week-old seedlings were used for VIGIS assays and 4-week-old plants were used for analysis of gene expression in response to pathogen infection or hormone treatments.

### Hormone and Drought Stress Treatments

For treatments with defense signaling hormones, 4-week-old plants were foliar sprayed with 100 μM methyl jasmonate (MeJA), 100 μM 1-amino cyclopropane-1-carboxylic acid (ACC) or 100 μM salicylic acid (SA) in 0.1% ethanol and with equal volume of 0.1% ethanol solution as mock controls. Drought stress was applied to the plants by withholding watering for 2 weeks and stress phenotype was recorded and photographed. Fully expanded leaves were detached from 10 individual plants and subjected to measure the rate of water loss according to previously described method (Liu et al., 2014). Roots from 10 plants were cut, cleaned and dried in 70°C oven for 24 h and the weight was calculated.

### Pathogen Inoculation and Disease Assays

Pathogen inoculation, disease assays with *Pst* DC3000 or with *B. cinerea* strain BO5-10 (provided by Dr. Tesfaye Mengiste, Purdue University, USA) and measurement of *in planta* pathogen growth were performed basically according to previously described protocols (AbuQamar et al., 2008; Li et al., 2014). Briefly, plants were inoculated by vacuum infiltration with *Pst* DC3000 in 10 mM MgCl_2_ solution (OD_600_ = 0.0002) or foliar spraying with *B. cinerea* spore suspension in 0.4% maltose solution (2 × 10^5^ spores/mL). Mock-inoculation control plants were treated by the same protocols with corresponding solutions without bacteria or spores. Leaf samples were collected at indicated time points after inoculation and used for gene expression, physiological and biochemical analyses.

### Characterization and Cloning of *SlSAHH* Genes

Tomato genome database at the SOL Genomics Network (SGN, http://solgenomics.net) was searched using BlastP program with characterized *Arabidopsis* AtSAHHs as queries and the predicted nucleotide and amino acid sequences for SlSAHHs were downloaded. EST (UniGene) and full-length cDNAs were obtained through searching against the tomato genome database and NCBI GenBank database, respectively, using predicted nucleotide sequences. Gene-specific primers for each *SlSAHH* gene were designed based on the predicted and putative EST and full-length cDNAs. The open reading frames (ORF) of the *SlSAHH* genes were PCR amplified from tomato cDNAs and cloned into pMD19-T vector by T/A cloning, yielding plasmids pMD19-SlSAHH1, pMD19-SlSAHH2, and pMD19-SlSAHH3. After confirmation by sequencing, these recombinant plasmids were used for further experiments.

### Vector Construction and VIGS Agroinfiltration

Fragments of 200–232 bp in sizes for *SlSAHHs* were amplified from plasmids pMD19-SlSAHH1, pMD19-SlSAHH2, and pMD19-SlSAHH3 using gene-specific primers (Supplementary Table S1) and cloned into TRV2 vector (Liu et al., 2002), yielding TRV2-SlSAHH1, TRV2-SlSAHH2, and TRV2-SlSAHH3. A 420 bp fragment, designated as SlSAHHa that corresponds to the conserved regions in ORFs of the *SlSAHH* genes, was amplified from pMD19-SlSAHH1 and cloned into TRV2 vector, yielding TRV2-SlSAHHa. The recombinant plasmids TRV2-SlSAHH1, TRV2-SlSAHH2, TRV2-SlSAHH3, and TRV2-SlSAHHa were introduced into *Agrobacterium tumefaciens* strain GV3101 by electroporation using GENE PULSER II Electroporation System (Bio-Rad Laboratories, Hercules, CA, USA). Agrobacteria carrying TRV2-SlSAHH1, TRV2-SlSAHH2, TRV2-SlSAHH3, TRV2-SlSAHHa or TRV2-GUS (a negative control plasmid with insertion of a GUS fragment) were grown in YEP medium containing 50 μg ml^-1^ rifampicin, 50 μg ml^-1^ kanamycin, and 25 μg ml^-1^ gentamicin for 24 h at 28°C with continuous shaking, collected by centrifugation and resuspended in infiltration buffer (10 mM MgCl_2_, 10 mM MES, 200 μM acetosyringone, pH5.7). Before virus-induced gene silencing (VIGS) assays, agrobacteria harboring TRV2-SlSAHH1, TRV2-SlSAHH2, TRV2-SlSAHH3, TRV2-SlSAHHa or TRV2-GUS were mixed with agrobacteria carrying TRV1 in a ratio of 1:1 and the bacterial concentration in suspension was adjusted to OD_600_ = 1.5. Agroinfiltration was performed by infiltration of agrobacterial suspension into the abaxial surface of 2-week-old seedlings using 1 ml needleless syringes (Liu et al., 2002). The agroinfiltrated plants were allowed to grow for 4 weeks and were then used for all experiments. In all VIGS assays, plants agroinfiltrated with agrobacteria harboring a TRV2-PDS (a gene encoding for phytoene desaturase) construct were always included to evaluate the efficiency of the VIGS protocol (Liu et al., 2002).

### *In Situ* Detection of H_2_O_2_ and Measurement of SAHH Activity

*In situ* detection of H_2_O_2_ was carried out by 3, 3-diaminobenzidine (DAB) staining (Thordal-Christensen et al., 1997; Li et al., 2014). Leaf samples were dipped into DAB (Sigma-Aldrich, St. Louis, MO, USA) solution (1 mg/mL, pH3.8) and incubated for 8 h in the dark at room temperature. Accumulation of H_2_O_2_ in stained leaves was visualized using a digital camera or observed under a Leica CTR5000 microscopy (Leica Microsystems, Hongkong, China). Measurement of SAHH activity in leaves was carried out based on a method described previously (Wolfson et al., 1986) using DTNB spectrophotometric assay kits (Roche, Shanghai, China). Briefly, ∼100 mg of leaf samples were ground in ice-cold HEPES buffer (pH7.8, 50 mM HEPES, 5 mM DTT, 1 mM Na_2_EDTA, 5 mM ascorbic acid, 10 mM boric acid, 20 mM Na-metabisulfate and 4% Polyvinylpyrrolidone) and the extracts were collected by centrifugation at 4°C for 5 min. The supernatants (150 μL) were passed by centrifugation for 15 s through Sephadex G25 columns to remove salts and other small molecules and the purified extracts were then used for enzyme activity assays. Reactions were conducted at 25°C for 15 min in reaction buffer containing 50 μM HEPES-KOH (pH7.5), 1 mM EDTA, 150 μM SAH, 100 μM 5, 5-dithio-bis(2-nitrobenzoic acid; DTNB) and 10 mg purified extracts. Reactions without SAH were used as reference controls for each corresponding sample. All reactions were measured spectrophotometrically for the absorbance at A412. Amounts of the reduced DTNB in the reactions were calculated using a molar extinction coefficient of 13600 M^-1^ cm^-1^. Protein concentrations in purified extract samples were determined using Bio-Rad protein assay kits (Bio-Rad, Hercules, CA, USA) following the recommended protocol.

### qRT-PCR Analysis of Gene Expression

Total RNA was extracted using Trizol regent (TaKaRa, Dalian, China) and treated with RNase-free DNase to erase DNAs according to the manufacturer’s instructions. First-strand cDNA was synthesized from 1 μg of total RNA by reverse transcription using the PrimeScript RT regent kit (TaKaRa, Dalian, China) according to the manufacturer’s protocol. qPCR was performed on a CFX96 real-time PCR system (BioRad, Hercules, CA, USA) and each reaction contained 12.5 μL SYBR Premix Ex TaqTM (TaKaRa, Dalian, China), 0.1 μg cDNA and 7.5 pmol of each gene-specific primer (Supplementary Table S1) in a final volume of 25 μL. Quantification of transcript levels of genes of interest related to transcript levels of a tomato actin gene was performed and the comparative Ct method for relative quantification was used to analyze data. Relative gene expression levels were calculated using 2^-ΔΔCT^ method and three independent biological replicates were analyzed.

### Measurement of SA Content

Extraction of SA from leaf samples was carried out using a mixed solid phase extraction method. Briefly, 200 mg leaf powder were extracted in 2 ml 80% methanol and kept in 4°C overnight. The samples were treated by centrifugation at 10000 × *g* for 10 min and the residues were re-extracted by 80% methanol. The supernatant was brought to a final volume of 10 ml in 33% methanol and 2% ammonia and then passed through Oasis columns (Waters, Milford, CO, USA) pre-eluted with 2 ml methanol and 2 ml 2% ammonia. SA in columns was first washed with methanol and 1% formic acid and then eluted by 2 ml 2% ammonia and 2 ml methanol. SA in samples was measured on Aglient 6400 LC-MS (Agilent, Palo Alto, CA, USA) and the SA content was calculated according to the formula (100 ng/mL × the area of the sample)/(the area of the standard × the volume)/the fresh weight.

### Detection of Callose Deposition

Collected leaf samples were treated in 50% ethanol, 25% phenol, and 25% lactic acid solution at 65°C for 30 min and then transferred to same solution. The samples were rinsed with distilled water for three times and transferred to staining solution (0.01% aniline blue in 150 mM K_2_HPO_4_, pH9.5) for 30 min. The stained leaves were detected in fluorescence microscope after balanced with PBS buffer.

### Experiment Design and Data Analysis

All experiments were repeated independently three times. At least 10 plants were included in each of independent experiments or samples from 10 individual plants were collected for analysis. Data obtained from three independent experiments were averaged and subjected to statistical analysis according to the Student’s *t*-test. The probability values of *p* < 0.05 were considered as significant difference between treatments and their corresponding controls.

## Results

### Characterization of SlSAHHs in Tomato

By Blastp searches against the tomato genome database using the characterized *Arabidopsis* AtSAHHs as queries, three predicted loci that encode putative SlSAHHs were obtained. For further convenience, we designated these putative SlSAHHs as *SlSAHH1* (Solyc12g098500), *SlSAHH2* (Solyc09g092380), and *SlSAHH3* (Solyc09g092390). ESTs and putative full-length cDNAs for *SlSAHHs* were identified in the tomato genome database and NCBI GenBank database, respectively, indicating that *SlSAHHs* are constitutively expressed in tomato. We cloned and sequenced the ORFs of *SlSAHHs* and the obtained ORF sequences of *SlSAHHs* are identical to the predicted ones. The SlSAHH proteins are 485 amino acids in size and all of them contain a SAHH (*S*-adenosylhomocysteine hydrolase) domain. Phylogenetic tree analysis revealed that the SlSAHH proteins showed >94% of sequence identity each other and ∼92% to *Arabidopsis* AtSAHH1 and AtSAHH2. Notably, SlSAHH2 and SlSAHH3 have 99% of identity.

### Expression Changes of *SlSAHHs* in Response to Pathogen Infection and Defense Hormone Treatments

**FIGURE 1:**
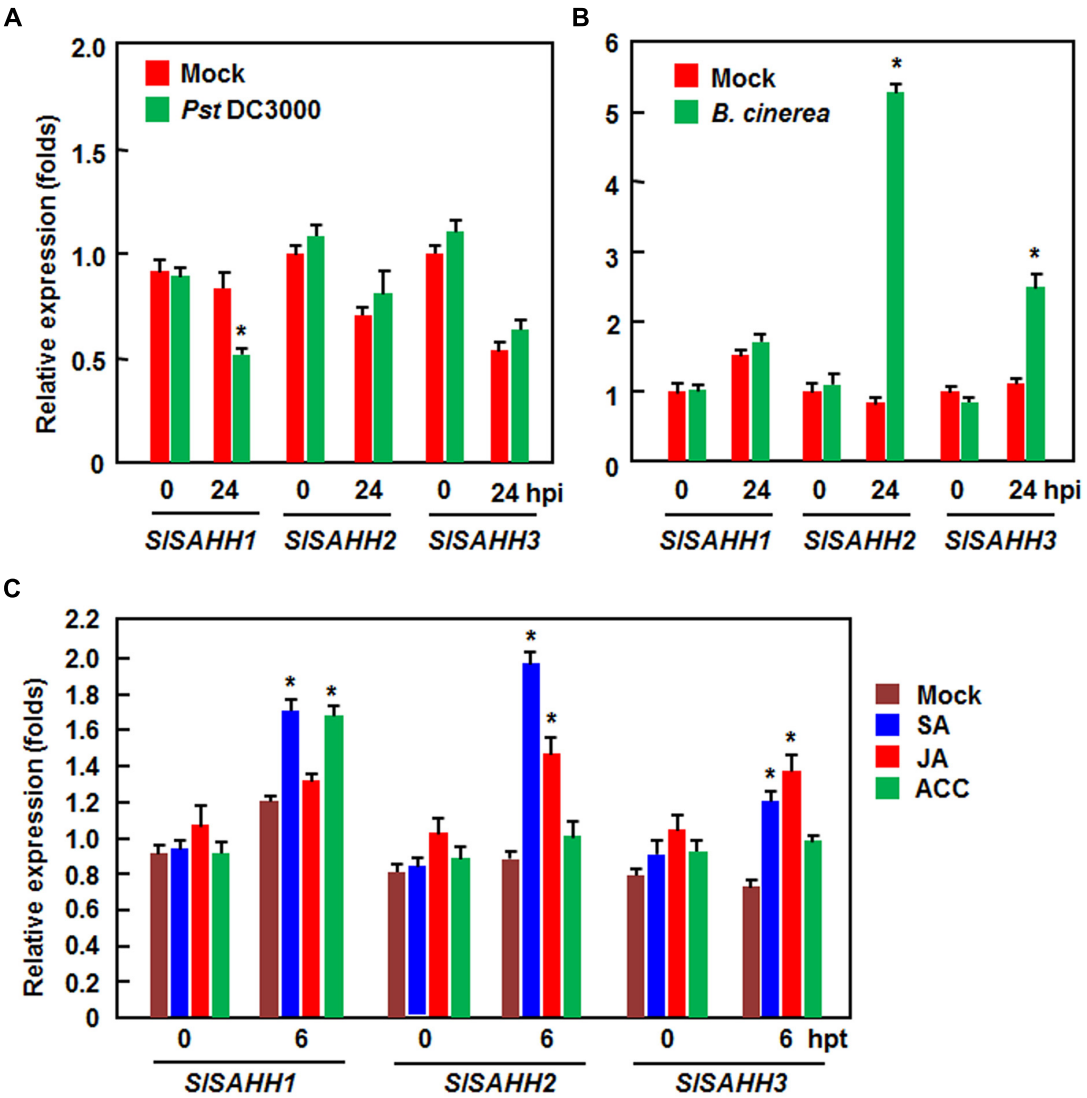
**Expression patterns of *SlSAHHs* in response to pathogens and defense hormones.** Four-week old plants were inoculated by vacuum infiltration with *Pseudomonas syringae* pv. *tomato* DC3000 (OD_600_ = 0.0002) **(A)**, foliar spraying with spore suspension (2 × 10^5^ spores/mL) of *B. cinerea* **(B)** or with similar volumes of the same solution as mock-inoculation controls. Four-week-old plants were treated by foliar spraying with 100 μM SA, 100 μM MeJA, 100 μM ACC or solution as a control **(C)**. Leaf samples were collected at indicated time points after inoculation or treatment. Expression data were normalized with the value of reference *Actin* gene and relative expression levels were shown as folds of the *Actin* expression level. Data presented are the means ± SD from three independent experiments and ^∗^ above the columns indicate significant differences at *p* < 0.05 level between the pathogen-inoculated or hormone-treated plants and the mock-inoculated/treated plants.

To explore the possible role of SlSAHHs in disease resistance, we analyzed the expression changes of *SlSAHHs* in response to infection by *Pst* DC3000 and *B. cinerea* as well as to treatments with three well-known defense signaling hormones such as SA, JA and ACC (a precursor or ET). In *Pst* DC3000-inoculated plants, the expression level of *SlSAHH1* was decreased significantly at 24 h after inoculation, whereas the expression of *SlSAHH2* and *SlSAHH3* was not affected (**Figure 1A**). By contrast, the expression of *SlSAHH2* and *SlSAHH3* in *B. cinerea*-inoculated plants were markedly induced, leading to 4.4 and 1.6 folds of increases over those in mock-inoculated control plants, respectively; however, the expression of *SlSAHH1* was not changed (**Figure 1B**). Meanwhile, we also analyzed the expression changes of *SlSAHHs* in response to different defense signaling hormones. As shown in **Figure 1C**, the expression of *SlSAHH1* and *SlSAHH3* was induced by SA at 6 h after treatment, but the expression of *SlSAHH2* was not affected. The expression of *SlSAHH1* and *SlSAHH2* was induced by JA and ACC at 6 h after treatment, but the expression of *SlSAHH3* was not affected (**Figure 1C**). These data indicate that *SlSAHHs* respond with distinct expression induction patterns to different pathogens and defense signaling hormones, suggesting possible involvements of SlSAHHs in defense response of tomato plants against pathogen infection.

### Co-Silencing of *SlSAHHs* Inhibited Vegetable Growth in Tomato

**FIGURE 2:**
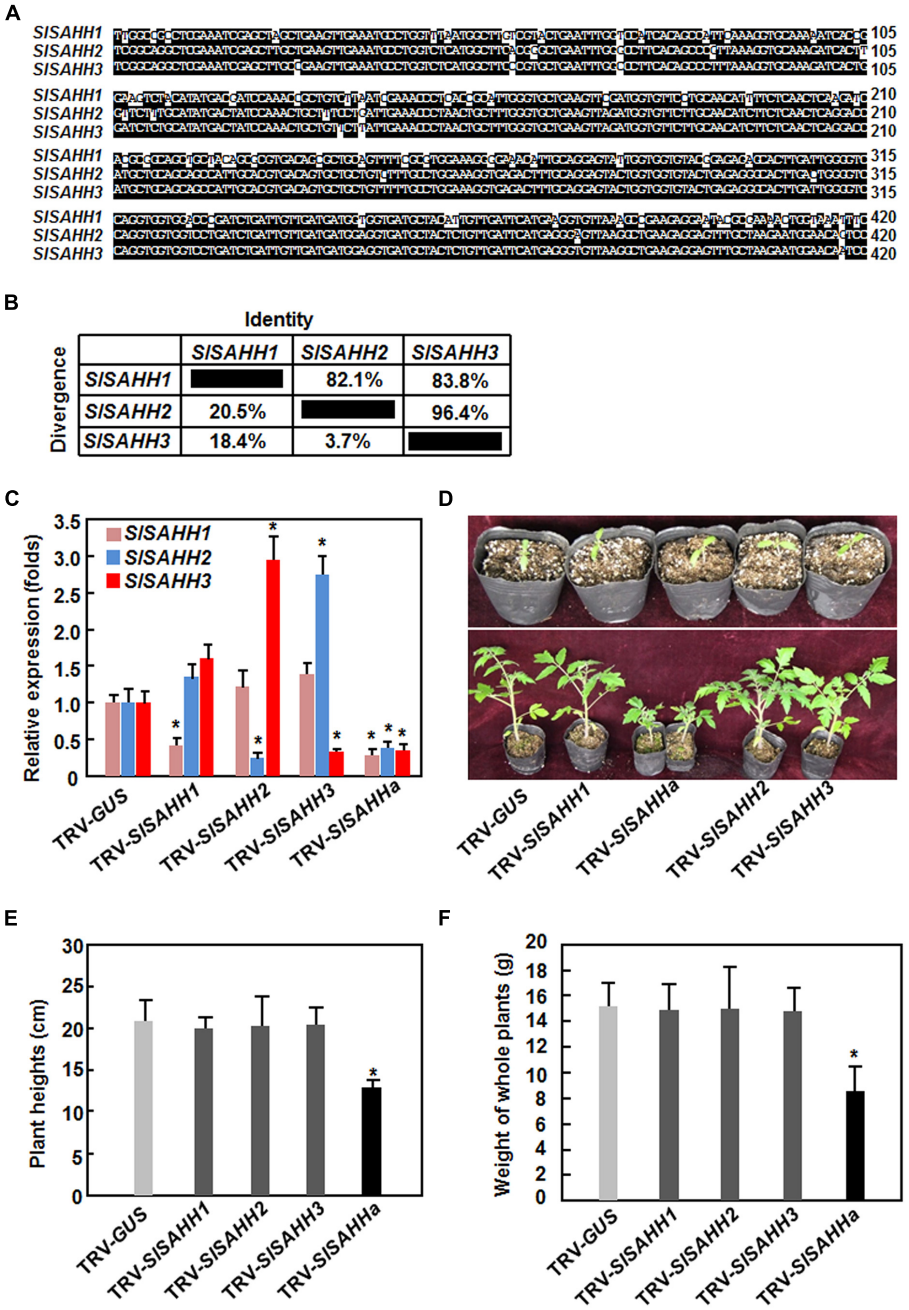
**Virus-induced gene silencing-based silencing efficiency and specificity for *SlSAHHs* and co-silencing of *SlSAHHs* resulted in abnormal growth phenotypes.** Alignment **(A)** and the identity and divergence **(B)** of the VIGS sequences from *SlSAHHs* for co-silencing. The VIGS sequences were aligned by ClustalW program in DNAStar software and the identity and divergence percentages were obtained from the alignment. **(C)** Silencing efficiency and specificity. Ten-day-old seedlings were infiltrated with agrobacteria carrying TRV-SlSAHH1, TRV-SlSAHH2, TRV-SlSAHH3, TRV-SlSAHHa or TRV-GUS constructs and leaf samples were collected 3 weeks after agroinfiltration. Transcript levels for *SlSAHH* genes were analyzed by qRT-PCR using a tomato *SlActin* gene as an internal control. **(D)** Co-silencing of *SlSAHHs* inhibited vegetable growth in TRV-SlSAHHa-infiltrated plants. Upper row, seedlings at the time of agroinfiltration; Lower row, growth performance of the agroinfiltrated plants at 5 weeks after agroinfiltration. The experiments were repeated twice with similar results. **(E)** and **(F)** Plant heights and whole plant weights of the TRV-SlSAHH1-, TRV-SlSAHH2-, TRV-SlSAHH3-, TRV-SlSAHHa-, or TRV-GUS-infiltrated plants. Six-week-old plants were collected to measure the heights and weights at weeks after agroinfiltration. Data presented are the means ± SD from three independent experiments and ^∗^ above the columns indicate significant differences at *p* < 0.05 level between the expression levels of *SlSAHH* genes in *SlSAHH*-silenced and TRV-GUS-infiltrated non-silenced plants **(C)** or between the plant heights and weights in TRV-GUS- and TRV-SlSAHHa-infiltrated plants **(E)** and **(F)**.

To understand the biological functions of *SlSAHHs*, a series of VIGS-based functional analyses was carried out. For this purpose, specific fragments for *SlSAHH1, SlSAHH2*, and *SlSAHH3* were used to silence each of the individual *SlSAHH* genes. Considering that the SlSAHHs are highly conserved in amino acid sequences, a conserved fragment with high level of sequence identity among *SlSAHHs* (**Figure 2A**), designated as SlSAHHa, was also used to co-silence all *SlSAHH* genes. This SlSAHHa fragment was amplified from *SlSAHH1* and showed >82% of sequence identity to the corresponding regions of *SlSAHH2* and *SlSAHH3* (**Figure 2B**). The silencing efficiency and specificity were estimated by qRT-PCR analysis of the transcript levels of each *SlSAHH* gene at 3 weeks after VIGS infiltration when >90% of the *PDS*-silenced plants displayed bleaching symptom. In *SlSAHH1*-, *SlSAHH2*-, and *SlSAHH3*-silenced plants, the transcript levels of *SlSAHH1, SlSAHH2*, and *SlSAHH3* were decreased by 63, 65, and 65%, respectively, as compared with those in TRV-GUS-infiltrated non-silenced plants (**Figure 2C**). The expression levels of *SlSAHH2* and *SlSAHH3* in *SlSAHH2*-silenced plants were slightly increased (**Figure 2C**). However, the expression levels of *SlSAHH3* in *SlSAHH2*-silenced plants and *SlSAHH2* in *SlSAHH3*-silenced plants were upregulated by 2.6 and 2.3 folds, respectively, as compared with those in TRV-GUS-infiltrated non-silenced plants; while the expression of *SlSAHH1* in *SlSAHH2*- and *SlSAHH3*-silenced plants was not significantly affected (**Figure 2C**). These data imply that SlSAHH2 and SlSAHH3 may have functional redundancy. In the TRV-SlSAHHa-infiltrated plants, the expression levels of *SlSAHH1, SlSAHH2*, and *SlSAHH3* were simultaneously and significantly decreased by 68, 63, and 64%, respectively, as compared with those in TRV-GUS-infiltrated non-silenced plants (**Figure 2C**), indicating that SlSAHHa could co-suppress the expression of the *SlSAHH* genes in tomato.

During our repeated experiments, no abnormal growth phenotype was observed in *SlSAHH1*-, *SlSAHH2*-, and *SlSAHH3*-silenced plants (**Figures 2D,F**). However, co-silencing of *SlSAHH1, SlSAHH2*, and *SlSAHH3* significantly inhibited the vegetable growth of the TRV-SlSAHHa-infiltrated plants (**Figures 2D,F**), leading to decrease of 35% for plant heights (**Figure 2E**) and of 45% for whole plant weight (**Figure 2F**) as compared to those of the TRV-GUS-infiltrated non-silenced plants. These data indicate that functional SlSAHHs are required for normal vegetable growth in tomato.

### Co-Silencing of *SlSAHHs* Enhanced Resistance to *Pst* DC3000 but Not *B. cinerea*

**FIGURE 3:**
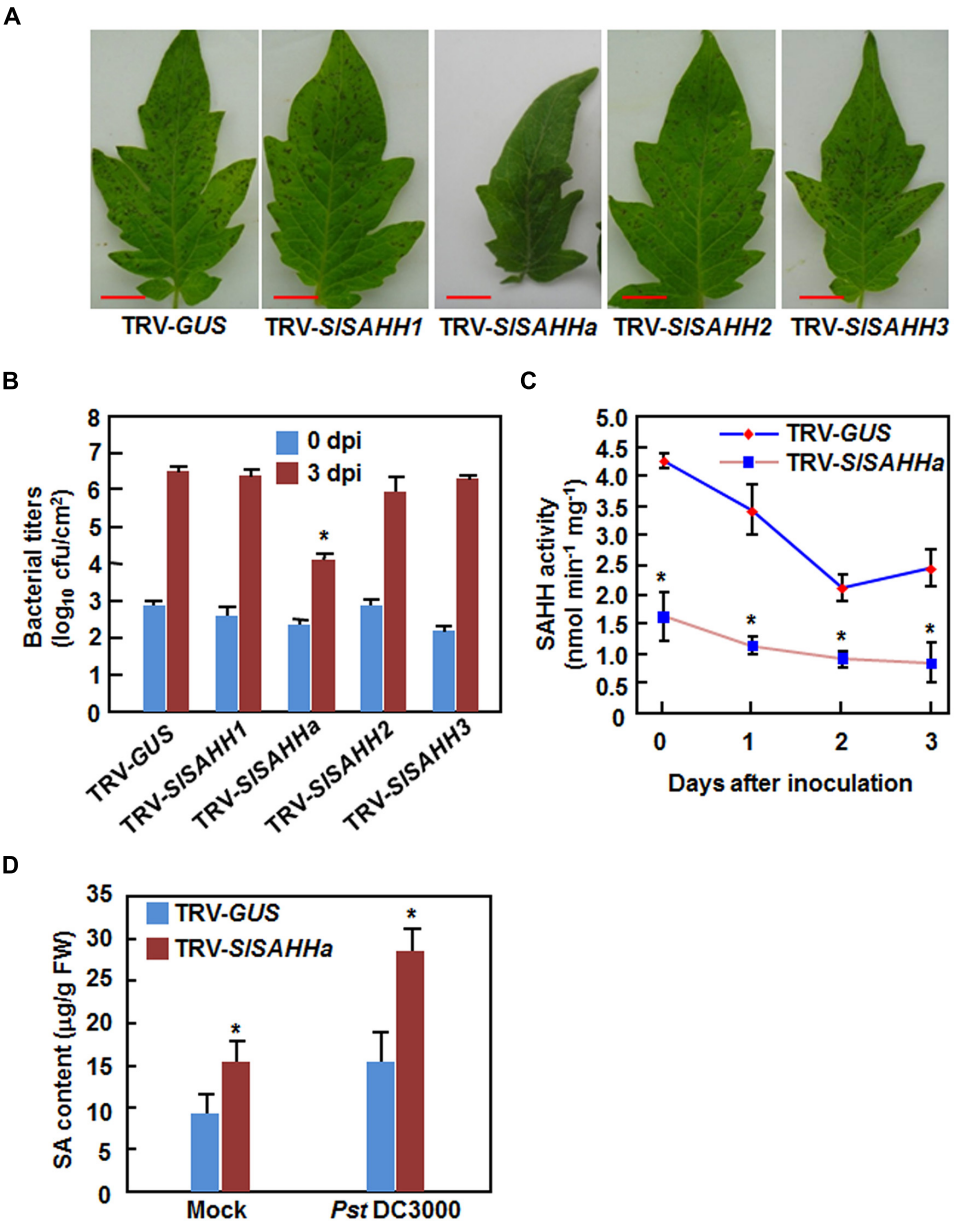
**Co-silencing of *SlSAHHs* conferred enhanced resistance to *P. syringae* pv. *tomato* DC3000.** Ten-day-old seedlings were infiltrated with agrobacteria carrying TRV-SlSAHH1/2/3, TRV-SlSAHHa or TRV-GUS constructs and were inoculated by vacuum infiltration with *Pst* DC3000 (OD_600_ = 0.0002) at 4 weeks after VIGS infiltration. **(A)** Representative disease symptom on leaves of the TRV-GUS- and TRV-SlSAHH-infiltrated plants. Photos were taken 3 days after inoculation (dpi). **(B)** Bacterial growth in inoculated leaves of TRV-GUS- and TRV-SlSAHH-infiltrated plants at 0 and 3 dpi. **(C)** Changes of SAHH activity in TRV-GUS- and TRV-SlSAHHa-infiltrated plants after inoculation with *Pst* DC3000. **(D)** SA contents in TRV-GUS- and TRV-SlSAHHa-infiltrated plants without and with inoculation with *Pst* DC3000. Leaf samples were collected at 24 h after inoculation with *Pst* DC3000 or with 10 mM MgCl_2_ as mock inoculation controls. Similar results were obtained in independent experiments **(A)** and data presented in **(B), (C)**, and **(D)** are the means ± SD from three independent experiments. ^∗^ above the columns in **(B), (C)**, and **(D)** indicate significant differences at *p* < 0.05 level between the TRV-SlSAHHa- and TRV-GUS-infiltrated plants.

To explore the involvement of *SlSAHHs* in disease resistance, we compared the disease phenotypes between *SlSAHH*-silenced and non-silenced plants after infection with *Pst* DC3000 or *B. cinerea*. Under our experiment condition, typical disease symptom was appeared at 4 days after inoculation (dpi) with *Pst* DC3000. At this time point, large numbers of small necrotic spots were seen in leaves of the *SlSAHH1*-, *SlSAHH2*-, *SlSAHH3*-silenced and the TRV-GUS-infiltrated control plants; however, almost no necrotic spot was observed in leaves of the TRV-SlSAHHa-infiltrated plants (**Figure 3A**). This was further confirmed by measurement of the bacterial growth *in planta*. At 4 dpi, the bacterial populations in leaves of the TRV-GUS-infiltrated control plants and of the *SlSAHH1*-, *SlSAHH2*-, *SlSAHH3*-silenced plants were comparable, accounting for 4.17 × 10^6^ CFU/cm^2^, 2.82 × 10^6^ CFU/cm^2^, 1.21 × 10^6^ CFU/cm^2^ and 1.73 × 10^6^ CFU/cm^2^, respectively (**Figure 3B**). However, the bacterial population in leaves of the TRV-SlSAHHa-infiltrated plants at 4 dpi was 1.15 × 10^4^ CFU/cm^2^, giving ∼360 times lower than that in leaves of the TRV-GUS-infiltrated plants (**Figure 3B**). Additionally, we also analyzed the changes in SAHH activity in TRV-GUS- and TRV-SlSAHHa-infiltrated plants after infection of *Pst* DC3000. As shown in **Figure 3C**, the SAHH activity in TRV-SlSAHHa-infiltrated plants was significantly reduced, accounting for 31% of the activity in the TRV-GUS-infiltrated plants at 0 h after inoculation. However, the SAHH activity in both of the TRV-GUS- and TRV-SlSAHHa-infiltrated plants was decreased with the times during a period of 3 dpi and the activity in TRV-SlSAHHa-infiltrated plants was decreased significantly, as compared with those in the TRV-GUS-infiltrated plants at 1, 2, and 3 dpi (**Figure 3C**). We also examined whether co-silencing of *SlSAHHs* affected the endogenous SA levels. As shown in **Figure 3D**, the SA level in TRV-SlSAHHa-infiltrated plants was significantly increased by 52% compared to that in TRV-GUS-infiltrated plants without inoculation of *Pst* DC3000; however, the SA level in TRV-SlSAHHa-infiltrated plants showed a further increase of 85% as compared to that in TRV-GUS-infiltrated plants at 24 h after inoculation with *Pst* DC3000 (**Figure 3D**). These data indicate that co-silencing of *SlSAHHs* resulted in an enhanced resistance of tomato plants to *Pst* DC3000 as revealed by the reduced disease symptom, decreased bacterial population, increased SA level and suppressed the SAHH activity.

**FIGURE 4:**
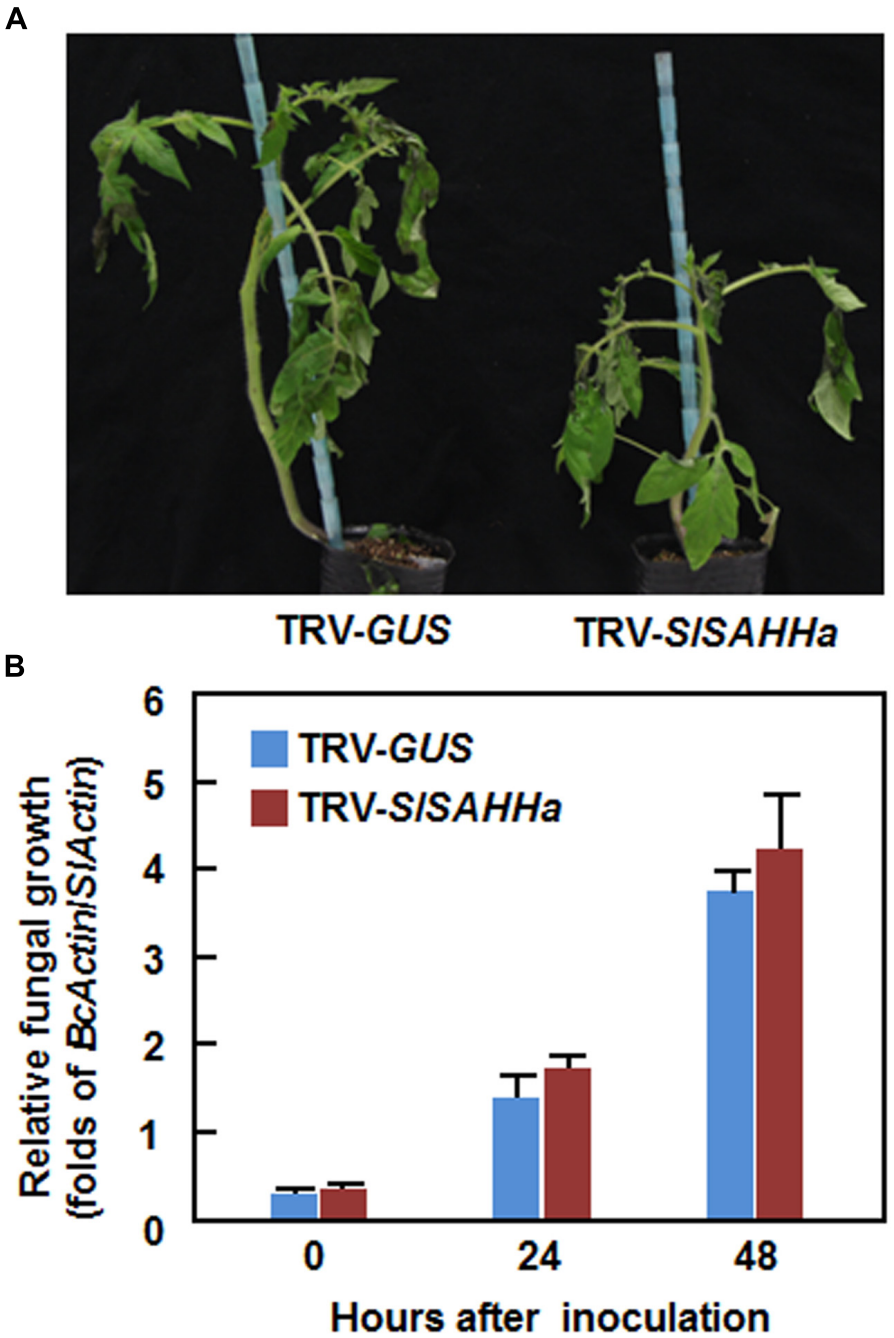
**Co-silencing of *SlSAHHs* did not affect the resistance to *Botrytis cinerea*.** Ten-day-old seedlings were infiltrated with agrobacteria carrying TRV-SlSAHHa or TRV-GUS construct and were inoculated by foliar spraying spore suspension (2 × 10^5^ spores/mL) of *B. cinerea* at 4 weeks after VIGS infiltration. **(A)** Representative disease symptom on *B. cinerea*-inoculated TRV-GUS- and TRV-SlSAHHa-infiltrated plants. Photos were taken at 4 days after inoculation. **(B)** *In planta* growth of *B. cinerea* in TRV-GUS- and TRV-SlSAHHa-infiltrated plants. Transcript levels for *B. cinerea BcActinA* and tomato *SlActin* genes in *B. cinerea*-inoculated plants were analyzed using qRT-PCR and *in planta* fungal growth was shown as ratios of transcript levels of *BcActinA*/*SlActin*. Similar results were obtained in independent experiments **(A)** and data presented in **(B)** are the means ± SD from three independent experiments. No significant differences at *p* < 0.05 level was detected in fungal growth between the TRV-SlSAHHa- and TRV-GUS-infiltrated plants.

We next examined whether co-silencing of *SlSAHHs* also affected resistance to *B. cinerea*, a necrotrophic fungal pathogen that has distinct infection style from that of *Pst* DC3000. In the *B. cinerea*-inoculated plants, disease symptom was seen at 2 dpi and the diseased leaves drooped (**Figure 4A**). However, no significant difference in appearance and symptom of *B. cinerea*-caused disease was observed between the TRV-SlSAHHa- and TRV-GUS-infiltrated plants (**Figure 4A**). Further, the *in planta* fungal growth, represented by ratios of *B. cinerea AcActin*/tomato *SlActin* transcripts, in leaves of TRV-SlSAHHa-infiltrated plants was comparable to those in leaves of TRV-GUS-infiltrated plants at 1 and 2 dpi (**Figure 4B**). These results indicate that co-silencing of *SlSAHHs* did not affect the resistance against *B. cinerea* in tomato.

### Co-Silencing of *SlSAHHs* Conferred a Constitutively Activated Immune Response

**FIGURE 5:**
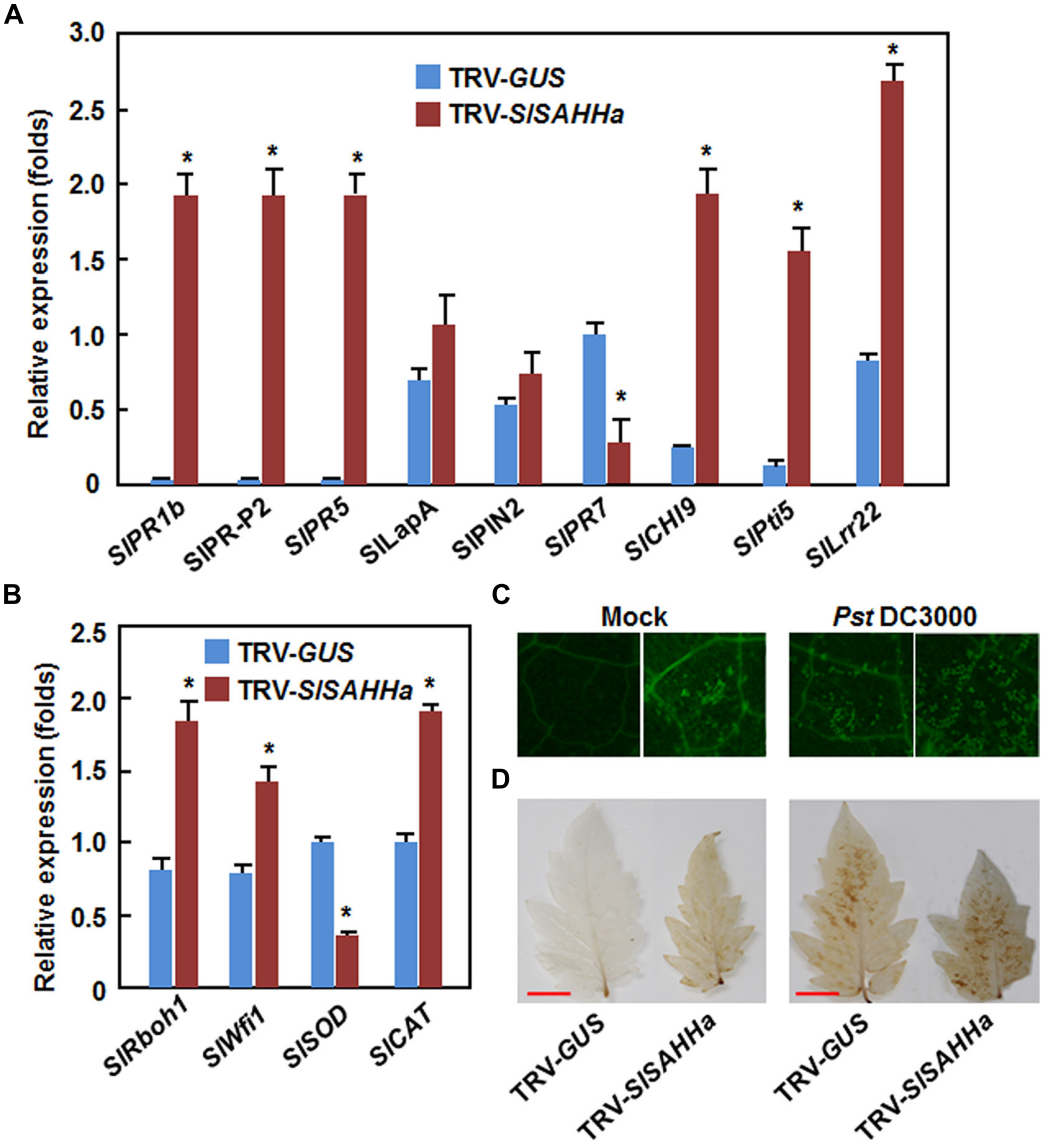
**Co-silencing of *SlSAHHs* conferred a constitutively activated immune response.** Ten-day-old seedlings were infiltrated with agrobacteria carrying TRV-SlSAHHa or TRV-GUS construct and leaf samples were collected at 4 weeks after agroinfiltration for analyzing expression of defense-related genes and staining of H_2_O_2_ accumulation. **(A)** Expression patterns of defense-related and PTI marker genes in TRV-GUS- and TRV-SlSAHHa-infiltrated plants. **(B)** Expression patterns of ROS-generating and scavenging genes in TRV-GUS- and TRV-SlSAHHa-infiltrated plants. **(C)** and **(D)** Callose deposition and H_2_O_2_ accumulation in TRV-GUS- and TRV-SlSAHHa-infiltrated plants without or with inoculation with *Pst* DC3000. Leaf samples were collected at 24 h after inoculation with *Pst* DC3000 or with 10 mM MgCl_2_ as mock inoculation controls. Data presented in **(A)** and **(B)** are the means ± SD from three independent experiments and ^∗^ above the columns indicate significant differences at *p* < 0.05 level between the TRV-SlSAHHa- and TRV-GUS-infiltrated plants. Similar results in **(C)** and **(D)** were obtained in independent experiments.

To gain insight into the mechanism of the enhanced *Pst* DC3000 resistance in TRV-SlSAHHa-infiltrated plants, we examined and compared the expression patterns of some well-known defense-related and PAMP-triggered immunity (PTI) marker genes in TRV-GUS- and TRV-SlSAHHa-infiltrated plants. As shown in **Figure 5A**, the expression levels of *SlPR1b, SlPR-P2*, and *SlPR5*, three defense-related genes that are thought to be regulated through the SA-mediated signaling pathway, and *SlCHI9*, a known defense-related gene, were dramatically upregulated in the TRV-SlSAHHa-infiltrated plants, giving >100 folds of increases for *SlPR1b, SlPR-P2*, and *SlPR5* and 8.2 folds for *SlCHI9* over those in the TRV-GUS-infiltrated plants. However, the expression levels of *SlLapA* and *SlPIN2*, two defense-related genes that are believed to be modulated via the JA-ET signaling pathway, were comparable to those in the TRV-GUS-infiltrated plants (**Figure 5A**). By contrast, the expression level of *SlPR7*, another JA/ET signaling pathway-regulated defense-related gene, in TRV-SlSAHHa-infiltrated plants was significantly downregulated by 2.2 folds as compared with that in the TRV-GUS-infiltrated plants (**Figure 5A**). Notably, the expression of *SlPti5* and *SlLrr22*, two PTI marker genes in tomato (Taylor et al., 2012), in TRV-SlSAHHa-infiltrated plants were markedly upregulated, leading to 7.3 and 2.8 folds of increases over those in TRV-GUS-infiltrated plants (**Figure 5A**). Furthermore, the expression levels of *SlRbohB* and *SlWfi1*, two genes for NADPH oxidases involved in generation of ROS (Sagi et al., 2004), and *SlCAT*, a gene for catalase involved in scavenging of H_2_O_2_, were significantly increased, while the expression of *SlSOD*, a gene for superoxide dismutase involved in scavenging of superoxide anion, was downregulated in TRV-SlSAHHa-infiltrated plants, as compared with those in TRV-GUS-infiltrated plants (**Figure 5B**). These results indicate that co-silencing of *SlSAHHs* led to upregulated expression of some SA signaling pathway-modulated defense-related genes, PTI-related genes and ROS-related genes.

To further confirm the PTI responses in TRV-SlSAHHa-infiltrated plants, we analyzed the patterns of callose deposition and *in situ* ROS accumulation in leaves of the TRV-SlSAHHa- and TRV-GUS-infiltrated plants without or with inoculation with *Pst* DC3000. Without inoculation with *Pst* DC3000, As shown in, no significant staining of callose deposition and H_2_O_2_ accumulation in leaves of the TRV-GUS-infiltrated plants was observed; however, obvious callose deposition and H_2_O_2_ accumulation was detected in leaves of the TRV-SlSAHHa-infiltrated plants (**Figures 5C,D**). Furthermore, after inoculation with *Pst* DC3000, the callose deposition and H_2_O_2_ accumulation in leaves of the TRV-SlSAHHa-infiltrated plants were much more than those in leaves of the TRV-GUS-infiltrated plants (**Figures 5C,D**). These data indicate that co-silencing of *SlSAHHs* potentiates the *Pst* DC3000-induced PTI responses in TRV-SlSAHHa-infiltrated plants.

### Co-Silencing of *SlSAHHs* Enhanced Drought Stress Tolerance

**FIGURE 6:**
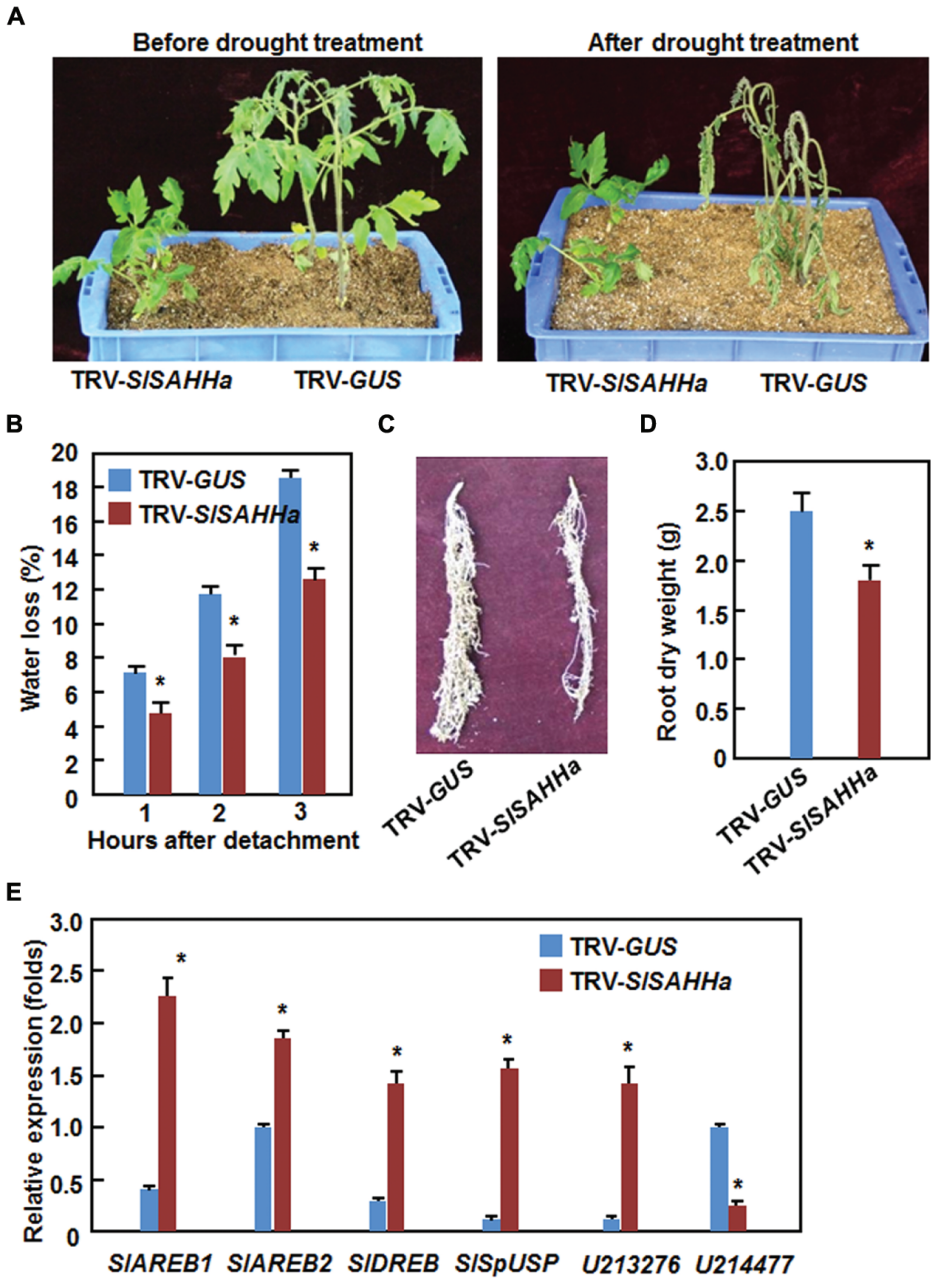
**Co-silencing of *SlSAHHs* conferred an enhanced drought stress tolerance.** Ten-day-old seedlings were infiltrated with agrobacteria carrying TRV-SlSAHHa or TRV-GUS construct and drought stress was applied to the plants by withholding water at 4 weeks after agroinfiltration. **(A)** Growth performance and drought phenotype of the TRV-GUS- and TRV-SlSAHHa-infiltrated plants before and after drought stress treatment. **(B)** Rates of water loss in detached leaves of the TRV-GUS- and TRV-SlSAHHa-infiltrated plants. **(C)** Root system of the TRV-GUS- and TRV-SlSAHHa-infiltrated plants. **(D)** Dry weight of roots from the TRV-GUS- and TRV-SlSAHHa-infiltrated plants. **(E)** Expression patterns of drought stress-related genes in TRV-GUS- and TRV-SlSAHHa-infiltrated plants before and after drought stress treatment. Similar results were obtained in independent experiments **(A,C)** and data presented in **(B), (D)**, and **(E)** are the means ± SD from three independent experiments and ^∗^ above the columns in **(B), (D)**, and **(E)** indicate significant differences at *p* < 0.05 level between the TRV-SlSAHHa- and TRV-GUS-infiltrated plants.

During our experiments toward on the functions of SlSAHHs in disease resistance, we occasionally noted that the TRV-SlSAHHa-infiltrated plants were not easier, as compared with the TRV-GUS-infiltrated plants, to appear wilting symptom when the plants were not watered during a 3-day period, indicating a possible role for SlSAHHs in drought stress tolerance. We thus examined whether *SlSAHHs* play a role in drought stress tolerance by analyzing and comparing the drought tolerance of the TRV-SlSAHHa- and TRV-GUS-infiltrated plants after withholding water for 2 weeks. As shown in **Figure 6A**, the growth status of the TRV-SlSAHHa- and TRV-GUS-infiltrated plants was similar before withholding water although the TRV-SlSAHHa-infiltrated plants were shorter than the TRV-GUS-infiltrated plants. At 2 weeks after withholding water, leaves of the TRV-GUS-infiltrated plants became curly and drooped and the plants wilted and eventually died; however, the TRV-SlSAHHa-infiltrated still grew well and showed normal appearance without any wilted leaves (**Figure 6A**). To confirm this observation, we analyzed the rate of water loss in detached leaves from the TRV-SlSAHHa- and TRV-GUS-infiltrated plants. The rate of water loss in leaves from the TRV-SlSAHHa-infiltrated plants was significantly decreased, leading to ∼30% of reduction, as compared with that in leaves from the TRV-GUS-infiltrated inoculated plants during a period of 3 h after detachment (**Figure 6B**). We also examined whether co-silencing of *SlSAHHs* affected the development of root system in tomato plants. Unexpectedly, the TRV-SlSAHHa-infiltrated plants had relatively small root system (**Figure 6C**) and the dry weight of the roots from the TRV-SlSAHHa-infiltrated plants was significantly decreased by 32% (**Figure 6D**), as compared with those of the TRV-GUS-infiltrated plants. We further examined and compared the expression patterns of some known drought stress-responsive genes in TRV-GUS- and TRV-SlSAHHa-infiltrated plants. In the TRV-SlSAHHa-infiltrated plants, the expression levels of *SlAREB1* (abscisic acid-responsive element bingding protein 1), *SlAREB2* (abscisic acid-responsive element bingding protein 2), *SlDREB* (dehydration-responsive element-binding protein), *SlSpUSP* and *SGN-U213276*, which are drought stress-upregulated genes (Gong et al., 2010; Orellana et al., 2010; Li et al., 2012; Loukehaich et al., 2012), were significantly increased by 4–7 folds for *SlAREB1, SlDREB, SlSpUSP* and *SGN-U213276* and onefold for *SlAREB2*, while the expression level of *SGN-U214777*, a drought stress-downregulated gene (Gong et al., 2010), was decreased by threefolds, as compared with those in TRV-GUS-infiltrated plants (**Figure 6E**). These data indicate that co-silencing of *SlSAHHs* led to an increased drought stress tolerance in tomato.

## Discussion

The functions of SAHHs, as targets of gene silencing suppressors, in interactions between viruses and their host plants were recently reported (Masuta et al., 1995; Yang et al., 2011; Cañizares et al., 2013). Several lines of indirect evidence from the altered expression of *SAHH* genes induced by pathogen infection and elicitor treatment (Kawalleck et al., 1992; Arasimowicz-Jelonek et al., 2013) led us to hypothesize that SAHHs should play a role in defense response to other pathogens. The present study provides direct experimental evidence that integrates the biological functions of SAHHs into plant stress responses, in addition to the previously reported functions in growth and development.

The tomato genome contains three *SAHH* genes, *SlSAHH1, SlSAHH2*, and *SlSAHH3*, while there are two *SAHH* genes in *Arabidopsis* (Rocha et al., 2005; Pereira et al., 2007; Li et al., 2008). Among the *SlSAHH* genes, *SlSAHH2* and *SlSAHH3*, showing 95.5% of identity in the ORFs, seem to be evolved through tandem duplication events as they distribute in tandem on the same location of chromosome 9. All of the tomato SlSAHHs and *Arabidopsis* AtSAHHs show ∼92% of identity between SlSAHHs and AtSAHHs, >94% among three SlSAHHs and 96% between two AtSAHHs (Rocha et al., 2005). High levels of sequence similarity and identity among tobacco NtSAHHs and among NtSAHHs, AtSAHHs and rice OsSAHH was also observed (Heim and Jelesko, 2004). Thus, it is likely that the plant SAHH proteins are quite conserved in sequences. However, the function modes of SlSAHHs and AtSAHHs seem to be different to some extent. For example, mutations in *Arabidopsis* AtSAHH1 resulted in abnormalities in growth and development (Furner et al., 1998; Rocha et al., 2005; Wu et al., 2009), whereas silencing of individual *SlSAHH* gene did not exhibit any defect in growth of the tomato plants. This is likely a consequence of functional redundancy among SlSAHHs. Particularly, the functional redundancy between *SlSAHH2* and *SlSAHH3* is much evident because of (1) high level of sequence similarity, (2) significant compensatory upregulated expression of *SlSAHH2* and *SlSAHH3* in plants that were silenced for *SlSAHH3* and *SlSAHH2*, respectively, and (3) similar expression patterns in response to *Pst* DC3000 and *B. cinerea*. Alternatively, silencing of individual *SlSAHH* gene may not significantly decrease the SAHH enzyme activity and thus cannot lead to a clear phenotype in growth alteration.

The total activity of SAHHs in TRV-SlSAHHa-infiltrated plants accounted for ∼30% of that in the non-silenced plants. The approach with co-silencing of *SlSAHHs* in the represent study is somewhat similar to the application of a chemical inhibitor of SAHHs that caused significant alterations in flower morphology of the tobacco plants (Fulneček et al., 2011), which have 4 *NtSAHH* genes (Heim and Jelesko, 2004). The facts that co-silencing of *SlSAHHs* resulted in significant growth inhibition and small root system demonstrate the requirement of SlSAHHs in vegetable growth and root development of tomato plants. This is in agreement with the previously observed stunted growth phenotype in the *Arabidopsis* and tobacco plants with reduced expression levels of *SAHH* genes due to mutations or antisense suppression (Tanaka et al., 1997; Li et al., 2008). Because the present study was mainly focused on the involvement of SlSAHHs in stress response, whether co-silencing of *SlSAHHs* has effects on reproductive developments (e.g., flower morphology and fertility) in tomato needs to be examined.

The observation that the *SlSAHHs*-co-silenced plants displayed an increase resistance to *Pst* DC3000 clearly demonstrates an important role for SlSAHHs in regulating immune response against pathogens. Firstly, the expression of *SlSAHH1* was significantly downregulated and accordingly, the SAHH activity was also decreased in both the SlSAHHs-co-silenced and non-silenced plants after infection by *Pst* DC3000, implying that suppression of the SAHH activity may be required for effectively activation of defense response upon infection of *Ps*t DC3000. Some viral gene silencing suppressors were shown to interact with SAHH and suppress its enzymatic activity (Yang et al., 2011; Cañizares et al., 2013). Secondly, the *SlSAHHs*-co-silenced plants with reduced expression levels of *SlSAHHs* and decreased activity of SAHH exhibited a constitutively activated defense response and *Pst* DC3000-induced PTI responses, as revealed by the elevated endogenous SA level, upregulated expression of some defense-related and PTI marker genes and increased callose deposition and ROS accumulation. It was previously shown that reduced SAHH activity due to mutations in *Arabidopsis* led to the DNA hypomethylation status and altered expression of genes involved in specific pathways (Jordan et al., 2007; Li et al., 2008; Ouyang et al., 2012). It is thus likely that the upregulated expression of defense-related and PTI marker genes in the *SlSAHHs*-co-silenced plants may be due to changes in DNA methylation status caused by reduced activity of SAHH. The increased *Pst* DC3000-induced PTI responses such as increased callose deposition and ROS accumulation in the *SlSAHHs*-co-silenced plants is similar to the observations that an active demethylation process is part of a mechanism to potentially act pathogen-induced immune response (Dowen et al., 2012; Yu et al., 2013).

However, co-silencing of *SlSAHHs* did not affect the resistance to *B. cinerea*, indicating that the requirement of SAHHs in defense response depends on pathogens. Distinct infection styles in and interaction nature with their host plants for *Pst* DC3000 and *B. cinerea* may account for the differential involvement of SAHHs in defense responses against these two different types of pathogens, one (hemi)biotrophic bacterial pathogen and one necrotrophic fungal pathogen (Glazebrook, 2005; Mengiste, 2012). In fact, there are also some differences in induction patterns of *SlSAHH* expression by *Pst* DC3000 and *B. cinerea* and the expression patterns of defense-related genes in *SlSAHHs*-co-silenced plants. Generally, defense responses against *Pst* DC3000 and *B. cinerea* are thought to be mediated by SA and JA/ET-dependent signaling pathways, respectively (Glazebrook, 2005). Whereas *Pst* DC3000 suppressed the expression of *SlSAHH1* but did not affect the expression of *SlSAHH2* and *SlSAHH3, B. cinerea* induced the expression of *SlSAHH2* and *SlSAHH3* but did not the expression of *SlSAHH1*. In agreement with this common knowledge, the expression of several SA-dependent signaling pathway-regulated defense-related genes such as *SlPR1b, SlPR-P2* and *SlPR5* was constitutively upregulated while the expression of the JA/ET-dependent signaling pathway-regulated defense-related genes was not affected for *SlLapA* and *SlPIN2* or even downregulated for *SlPR7* in *SlSAHHs*-co-silenced plants. Another, it was found that mutations in *Arabidopsis AtSAHH1* and antisense inhibition of *NtSAHH* in tobacco led to an increased content of cytokinins (Masuta et al., 1995; Li et al., 2008), a well-known growth hormone that is thought to play a role in defense response against *Pst* DC3000 but not affect the resistance to *B. cinerea* (Choi et al., 2010). It will be of interest to examine whether cytokinins are involved in the regulation of defense response in *SlSAHHs*-co-silenced plants. On the other hand, the involvement of SAHHs in defense response against other necrotrophic fungal pathogens such as *Alternaria brassicicola* and *Sclerotinia sclerotiorum* cannot be ruled out and thus needs to be further investigated.

The involvement of SAHHs in abiotic stress tolerance has not been defined yet. The observation that co-silencing of *SlSAHHs* resulted in increased drought stress tolerance demonstrates that SAHHs also play an important role in abiotic stress tolerance. Surprisingly, like the inhibition of aboveground vegetable growth, co-silencing of *SlSAHHs* also suppressed the root development leading to a small root system in *SlSAHHs*-co-silenced plants, consistent with that in the *Arabidopsis sahh1* mutant plants (Wu et al., 2009). Generally, a larger root system often provides better access to limited water in the soil environment and thus improves drought stress tolerance (Werner et al., 2010). Thus, it is unlikely that co-silencing of *SlSAHHs*-caused suppression of root system is responsible for the increased drought stress tolerance in *SlSAHHs*-co-silenced plants. By contrast, reduced rate of water loss, as revealed in the detached leaves, and enhanced drought stress response, as represented by the upregulated expression of some selected drought stress-responsive gene, might be the mechanisms that regulate the increased drought stress tolerance in *SlSAHHs*-co-silenced plants. It is thus speculated that co-silencing of *SlSAHHs* constitutively activates the stress responses and thereby enhanced drought tolerance in tomato.

## Conclusion

The present study was mainly focused on the biological function of SAHHs in regulating pathogen defense response in tomato. We found that co-silencing of three tomato *SlSAHH* genes confers increased immunity to *Pst* DC3000 and enhanced drought stress tolerance, demonstrating that, in addition to the previously reported involvement in plant growth and development, SAHHs also play important roles in regulating biotic and abiotic stress responses. However, several questions regarding the mechanism of action of SAHHs in biotic and abiotic stress response need to be addressed. Systematic studies on genome-wide profiling of gene expression and DNA methylome in *SlSAHHs*-co-silenced plants will help to identify genes that are affected by SlSAHHs and define their associations with specific pathways including those of the stress response pathways, providing insights into the molecular mechanism and the signaling pathways involved in SlSAHHs-regulated biotic and abiotic stress response. Another, further investigations on the metabolic changes, especially the dynamics of SAM/SAH ration, in *SlSAHHs*-co-silenced plants during biotic and abiotic stress responses will promote to elucidate the physiological and biochemical mechanisms for the actions of SlSAHHs in biotic and abiotic stress responses.

## Author Contributions

LH, YH, YZ, SL, DL, and HZ carried out most of the experiments. XL, HZ, and FS designed the experiments. HZ and FS drafted the manuscript and revised the manuscript with XL. All authors read and approved the final manuscript.

## Conflict of Interest Statement

The authors declare that the research was conducted in the absence of any commercial or financial relationships that could be construed as a potential conflict of interest.
