# Supplementary material for: Co-silencing of tomato S-adenosylhomocysteine hydrolase genes confers increased immunity against Pseudomonas syringae pv. tomato DC3000 and enhanced tolerance to drought stress
Source: Front Plant Sci. 2015 Sep 8;6:717. doi: 10.3389/fpls.2015.00717 (PMC4561804; doi:10.3389/fpls.2015.00717)
Supplement: Supplementary file 1 [file Table_1.DOC]

**Supplementary Table S1.** Primers used in this study for different purposes

| Primers | Sequences (5’-3’) | Size (bp) |
| --- | --- | --- |
| ***Cloning of cDNAs*** | | |
| SlSAHH1-F | CAATTTCTTCACTTTTTTCACTC | 1495 |
| SlSAHH1-R | TTAGTACCTGTAGTGAGCAGG |
| SlSAHH2-F | CTCAGATCTCATCTTAAACCC | 1640 |
| SlSAHH2-R | CACATTTAGGTCAACTTTCCAG |
| SlSAHH3-F | AAATCTCCCATTTTCTCTCAG | 1649 |
| SlSAHH3-R | GGTCTTTTTCAAACTTTCAGC |
| ***VIGS*** | | |
| SlSAHH1-VIGS-F | TGC TCTAGAAAAACAAATCCTAGCCGTCC | 205 |
| SlSAHH1-VIGS-R | CCG GAGCTCGTCCTTGACCTTGTACTCGC |
| SlSAHH2-VIGS-F | TGC TCTAGAGACGCTCACAGTGGAACAACG | 232 |
| SlSAHH2-VIGS-R | CCGGAGCTCGCTTTGTTTCCTTGAAGCAAATT |
| SlSAHH3-VIGS-F | TGC TCTAGAGGGAAGACAATTTGACAGTGG | 199 |
| SlSAHH3-VIGS-R | CCG GAGCTCGAAGGTAACTTGGAACACCATC |
| SlSAHHa-VIGS-F | TGCTCTAGA TCGGCAGGCTCGAAATCGAG | 420 |
| SlSAHHa-VIGS-R | CCGGAGCTC GGACTGTTCCATTCTTAGCA |
| ***qRT-PCR*** | | |
| SlSAHH1-qRT-F | CATTCCTCTGTGCCACTCTT | 97 |
| SlSAHH1-qRT-R | GATTAACGCCATGGGTAGAGAG |
| SlSAHH2-qRT-F | ACCGTCCATTTCCACAATCA | 132 |
| SlSAHH2-qRT-R | GGGTTTAAGATGAGATCTGAGAGG |
| SlSAHH3-qRT-F | GGGAAGACAATTTGACAGTGG | 88 |
| SlSAHH3-qRT-R | ACACACTATCATCCTAAACAAACTC |
| SlActin-qRT-F | CCAGGTATTGCTGATAGAATGAG | 113 |
| SlActin-qRT-R | GAGCCTCCAATCCAGACAC |
| SlPin2-qRT-F | CATCTTCTGGATTGCCCA | 106 |
| SlPin2-qRT-R | ACACACAACTTGATGCCCAC |
| SlLapA-qRT-F | GGGACTAATGATGTTTGGAA | 109 |
| SlLapA-qRT-R | GTGGCAATTTTATTTAGGCA |
| SlPR1b-qRT-F | TTTCCCTTTTGATGTTGCT | 96 |
| SlPR1b-qRT-R | TGGAAACAAGAAGATGCAGT |
| SlPRP2-qRT-F | CGATCTAAATTGATTTCATAGTACG | 116 |
| SlPRP2-qRT-R | TCGTGAAGGATATACAAAATACA |
| BcActin-qRT-F | CGTCACTACCTTCAACTCCATC | 107 |
| BcActin-qRT-R | CGGAGATACCTGGGTACATAGT |
| U213276-qRT-F | GTCAAACACTGGAAAGCATGAA | 110 |
| U213276-qRT-R | AGCTGCTCCACTTGTCTTATC |
| U214477-qRT-F | GGTGTTTGTGCTGACCTACT | 100 |
| U214477-qRT-R | CTTCCAAATCAGCCAAACCTTC |
| SlAREB1-RT-F | GTGGTGGGAAGGATGGAAATA | 120 |
| SlAREB1-RT-R | CTCTCACAACTCCAGCTCTAAC |
| SlAREB2-RT-F | CATGTGGTGAAGGTGGAAGA | 98 |
| SlAREB2-RT-R | CGCAGACTCCCTGTTCTTTAT |
| SlDREB-RT-F | CGGAGGAACTGGGTGAAATTA | 99 |
| SlDREB-RT-R | CGTCCACTGAATCACTGATCTT |
| SpUSP-RT-F | CGCGGCAAGAGAGAATACAT | 95 |
| SpUSP-RT-R | CTCTCATCGATAGCCACCATTATC |
| SlPR5-RT-F | GCTCGATTACGTCTTGTCTCTC | 104 |
| SlPR5-RT-R | CTCTAGCATGGTGGATTGACTT |
| SlPR7-RT-F | AAC TGC AGA ACA AGT GAA GG | 96 |
| SlPR7-RT-R | AAC GTG ATT GTA GCA ACA GG |
| SlCHI9-RT-F | AACGCGGGAATTGTTCGA | 112 |
| SlCHI9-RT-R | GCAGGACATGCGTCATTGTT |
| SlPti5-RT-F | ATTCGCGATTCGGCTAGACATGGT | 95 |
| SlPti5-RT-R | AGTAGTGCCTTAGCACCTCGCATT |
| SlLrr22-RT-F | AAGATTGGAGGTTGCCATTGGAGC | 100 |
| SlLrr22-RT-R | ATCGCGATGAATGATCGGTGGAGT |
| SlRboh1-RT-F | TGAGGAAGAGAAGCCCAATAAG | 91 |
| SlRboh1-RT-R | CACAAGACCAGAACCCAAATTC |
| SlWfi1-RT-F | AGGGAATGATAGAGCGTCG | 143 |
| SlWfi1-RT-R | CATCGTCATTGGACTTGGC |
| SlSOD-RT-F | GGC CAA TCT TTG ACC CTT TAT G | 183 |
| SlSOD-RT-R | AAG TCC AGG AGC AAG TCC AGT T |
| SlCAT-RT-F | CCC AGT TAA TGC TCC CAA GTG T | 118 |
| SlCAT-RT-R | AGG ACG ACA AGG ATC AAA CCT C |
